# Brief guide to western blot assays

**DOI:** 10.1016/j.mocell.2025.100297

**Published:** 2025-11-06

**Authors:** Seokjun G. Ha, Ji-Hoon Park, Mi-Young Kim, Seung-Jae V. Lee

**Affiliations:** Department of Biological Sciences, Korea Advanced Institute of Science and Technology, Daejeon 34141, South Korea

**Keywords:** Western blot

## Abstract

Western blot is an essential method that detects specific proteins using antibodies, which is one of the most widely applied techniques for protein detection. Here, we present a brief guide to western blot, following the overall procedure from the choice of antibodies to quantification. This guide will provide useful information for researchers who are unfamiliar with western blot assays.

## INTRODUCTION

**Figure 1:**
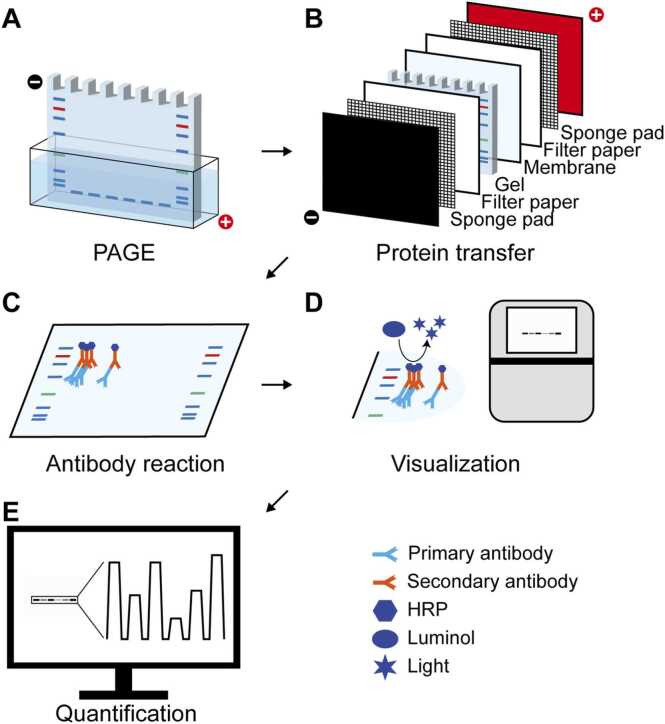
Overall flow of western blot. (A) The first step of western blot is performing polyacrylamide gel electrophoresis (PAGE). (B) Separated proteins in gel are then transferred to membrane. Transfer cassettes consist of gel and membrane that are sandwiched between filter papers and sponge pads. (C) The membrane is treated with primary antibody targeting proteins of interest, and subsequent secondary antibody targeting primary antibodies, conjugated with probes such as horseradish peroxidase (HRP) or a fluorophore. (D) Attached antibodies are typically detected using chemiluminescence. For chemiluminescence, HRP uses luminol as a substrate to generate light. (E) Band intensities from blot images are quantified using proper softwares.

Western blot is a widely used technique for the detection and quantification of proteins, crucial for assaying gene expression and regulation (Burnette, 1981, Renart et al., 1979; Jeong et al., 2018, Mahmood and Yang, 2012). Among various antibody–based protein quantification methods, such as microscopy and flow cytometry (Park et al., 2025, Song and Lee, 2024), western blot analysis has a unique advantage that provides information on the sizes of proteins (Begum et al., 2022). In addition, western blot is useful to investigate interactions between macromolecules and proteins in conjunction with coimmunoprecipitation and pulldown assays (Bai et al., 2016, DeCaprio and Kohl, 2020). Western blot steps include separation by polyacrylamide gel electrophoresis (PAGE), protein transfer, visualization, stripping, and quantification (Figure 1). Here, we provide a brief and practical guide for researchers who are unfamiliar with western blotting.

## SELECTION OF PROPER ANTIBODIES

A primary antibody is the most important reagent for western blot that directly binds the protein of interest. Researchers should consider diverse factors for the selection of primary antibodies, including specificity, host species, and the types of epitopes. Primary antibody is classified as monoclonal or polyclonal based on its specificity (Lipman et al., 2005). Monoclonal antibody is generated by a single clone of cells and targets a single epitope, while polyclonal antibody is a mixture of multiple antibodies that recognize multiple epitopes of the target protein. Although monoclonal antibodies generally have high specificity, they are not always the best choice because polyclonal antibodies are favorable to detect low-abundance or mutated proteins (Busby et al., 2016). Primary antibodies generally originate from mice or rabbits, and the choice of secondary antibody depends on the host species of the primary antibody. For the types of epitopes, linear epitopes and conformational epitopes are composed of sequentially and structurally consecutive amino acids, respectively. Thus, antibodies that target conformational epitopes may not properly recognize denatured proteins on a membrane (Forsstrom et al., 2015). Therefore, researchers should ensure that the antibody binds to the denatured protein. In cases where the antibody produces multiple bands, siRNA-mediated knockdown can be employed to identify the correct target band (Pillai-Kastoori et al., 2020a).

If proteins of interest do not originate from mammals, seeking proper antibodies for western blot is often challenging (Jeong et al., 2018). In this case, conjugating peptide of protein tags to the protein of interest will facilitate the detection because commercial antibodies targeting the popular tags usually have high affinity and specificity (Terpe, 2003, Yoon et al., 2024). The most widely used protein tags for cell biology are fluorescent proteins, which also enable visualization of proteins in live cells (Hutter, 2012). However, fluorescent protein tags are often not ideal for western blot analysis, because of their relatively large molecular weights. Alternatively, small peptide tags, such as FLAG, HA, and V5, which tend to minimally affect the sizes and functions of conjugated proteins, can be added to proteins of interest (DeCaprio and Kohl, 2020).

Secondary antibodies are probe-conjugated antibodies that target fragment crystallizable (Fc) regions of primary antibodies. Therefore, the prerequisite of the secondary antibodies is that they must bind Fc regions of the primary antibodies. Next, researchers should consider conjugates that enable the detection of proteins. Most secondary antibodies used for western blot are conjugated with peroxidase, such as horseradish peroxidase (HRP). The HRP-conjugated antibodies generate chemiluminescence by oxidizing luminescent materials using peroxide (Veitch, 2004). A fluorophore-conjugated antibody is also available for western blot and enables multiplexed detection of proteins on a single membrane using different colors of fluorophore (Gingrich et al., 2000, Schutz-Geschwender et al., 2004). However, the fluorescence-based detection is less sensitive and requires more expensive equipment than the chemiluminescence-based detection.

## POLYACRYLAMIDE GEL ELECTROPHORESIS

The first step of western blot is performing PAGE to separate proteins (Fig. 1A) (Laemmli and Favre, 1973, Weber and Osborn, 1969). Unlike DNA, complex structures and variations in net charge of proteins interfere with the separation of proteins based on their molecular weights. To resolve these issues, proteins are denatured using detergent, reducing agent, and high temperature. For the conventional Tris-glycine gel system, proteins are mixed with the Laemmli’s sample buffer that contains sodium dodecyl sulfate (SDS) and 2-mercaptoethanol or dithiothreitol (DTT). SDS is a detergent that denatures and evenly coats proteins with negative charge, and 2-mercaptoethanol or DTT is a reducing agent that cleaves disulfide bonds (Laemmli, 1970, Laemmli and Favre, 1973). The proteins are subsequently boiled at near 100°C for complete denaturation and separated with PAGE. The resolution of proteins in a gel depends on the concentration of acrylamide: low-percentage gels are used to separate high-molecular-weight proteins, whereas high-percentage gels are more suitable for low-molecular-weight proteins (Chrambach and Rodbard, 1971). Therefore, it is important to select the appropriate gel percentage to achieve optimal protein separation.

The Bis-Tris gel system is an advanced method for PAGE that utilizes neutral pH, which reduces unwanted protein degradation and enhances shelf time of gels (Penna and Cahalan, 2007). Furthermore, the Bis-Tris system provides two choices of running buffers to enhance resolution: 3-(N-morpholino)propanesulfonic acid (MOPS) or 2-(N-morpholino)ethanesulfonic acid (MES) running buffers. Due to the different mobility of MOPS and MES during electrophoresis, MOPS running buffer is suitable for separating large- to middle-sized proteins, and MES running buffer is suitable for separating relatively small proteins. For the Bis-Tris gel running, proteins are treated with lithium dodecyl sulfate sample buffer that contains 2-mercaptoethanol or DTT and heated at 70°C in advance (Penna and Cahalan, 2007).

## PROTEIN TRANSFER

After completing PAGE, proteins are transferred to a membrane to enable reaction with the protein of interest and antibodies (Fig. 1B). The most widely used materials for membranes are polyvinylidene fluoride (PVDF) and nitrocellulose (NC) (Tovey and Baldo, 1989, Towbin et al., 1979). The PVDF membrane requires activation using methanol before protein transfer because it is hydrophobic. PVDF membrane captures proteins more strongly and is more durable than the NC membrane (Xiang et al., 2021). The NC membrane exhibits relatively weak binding to proteins, but the NC membrane does not require activation because of its hydrophilicity and has low background signals.

For protein transfer to a membrane, wet transfer or semi-dry transfer is available. Wet transfer is a traditional way for protein transfer using a large volume of buffer that fully drowns transfer cassettes. Semi-dry transfer utilizes a minimal volume of buffer that barely wets transfer cassettes. Semi-dry transfer is quick and straightforward, but is not favorable to transfer large proteins and vulnerable to heat during transfer. Transfer cassettes consist of gel and membrane that are sandwiched between filter papers and outer sponge pads that keep the cassettes wet and tight (Mahmood and Yang, 2012, Towbin et al., 1979). Semi-dry transfer cassettes are similar to the wet transfer cassettes but do not contain sponge pads. Researchers should be aware of polarity during assembling transfer cassettes. Gels must be placed near the cathode because the proteins in gel coated with dodecyl sulfate carry a negative charge. Oppositely assembled cassettes will cause complete loss of proteins.

## VISUALIZING PROTEINS OF INTEREST

The next step is visualizing proteins of interest on the membrane. Blocking is the pretreatment of the membrane with proteins that do not bind the antibodies to prevent nonspecific binding of antibodies. In general, blocking solution consists of skim milk or bovine serum albumin that is dissolved in a proper buffer with a weak detergent (eg, phosphate-buffered saline with Tween 20 [PBST] or Tris-buffered saline with Tween 20 [TBST]) (Begum et al., 2022, Kim et al., 2023b, Liu et al., 2023). Membranes are soaked in the blocking solution with mild rocking. After blocking, primary antibodies are diluted in blocking solution to minimize the chances of nonspecific binding. The appropriate antibody concentration and incubation conditions (eg, duration and temperature) are typically provided in the manufacturer’s product sheet. The membranes are then washed with TBST multiple times to remove residual antibodies and treated with secondary antibodies dissolved in blocking solution or TBST. Commercial secondary antibodies usually have high specificity and affinity. Therefore, low concentrations of secondary antibodies are sufficient to bind primary antibodies. The membranes are ready to detect proteins of interest after secondary antibody treatment (Fig. 1C).

Detecting proteins of interest is achieved by chemical reaction if the secondary antibody is HRP-conjugated and by fluorescence if the secondary antibody is conjugated with a fluorophore (Gingrich et al., 2000, Huang and Amero, 1997, Song et al., 2023). For chemiluminescent detection, an enhanced chemiluminescence (ECL) substrate is utilized. The ECL substrate is separately supplied to two solutions: luminol/enhancer solution and peroxide solution. The enhancer stabilizes and amplifies signals from luminol oxidization, and the sensitivity of substrates is determined by enhancer (Diaz et al., 1995). However, highly sensitive substrates are generally accompanied by high background signals. Thus, researchers need to choose a substrate that provides proper signal intensity. Peroxide solution provides oxygen to oxidize luminol. The two solutions are mixed equally right before use and spread on the membrane, and luminescence is detected with an X-ray film or a chemiluminescence imaging system (Fig. 1D) (Begum et al., 2022, Kwak et al., 2024, Lee et al., 2024). For fluorescent detection, imaging system must be equipped with proper filter set, but no additional substrates are required. When using an imaging system, researchers can adjust exposure time and binning. Exposure time is defined as the duration of the sensor that is exposed to photons generated from samples. Therefore, longer exposure time results in a stronger signal. Signal accumulation mode is a useful option for obtaining optimal images, which captures a series of cumulative images during exposure time. Binning is the merging of signals in multiple pixels (Bass et al., 2017). For example, 3 × 3 binning is the merging of 9 pixels in a 3 × 3 square into one superpixel to enhance signal intensity. Therefore, higher binning is useful when signals from proteins of interest are dim, although it sacrifices the resolution of images.

## STRIPPING

Western blotting is a semiquantitative method that uses endogenous proteins that exhibit constant levels, such as glyceraldehyde 3-phosphate dehydrogenase (GAPDH), α-tubulin, and β-actin, as normalization controls (Le et al., 2024, Neris et al., 2021, Wang et al., 2023). Therefore, researchers often need to detect multiple proteins with a single membrane. Because multiplexed detection is limited for chemiluminescence method, stripping, a technique for removing attached antibodies from the used membrane, is important. Mild stripping utilizes weak detergents such as Tween 20 and low pH to detach antibodies. Mild stripping minimally affects proteins on the membrane, but antibodies with high affinity often remain on the membrane even after stripping. Harsh stripping utilizes strong detergents (eg, SDS), 2-mercaptoethanol, and high temperature (approximately 50°C), to completely remove antibodies. However, harsh stripping may cause significant loss of proteins on the membrane. The membranes are then washed with TBST multiple times to remove residual stripping solution that interferes with the antibody reaction. The membranes are ready for blocking after stripping (Yeung and Stanley, 2009).

## QUANTIFICATION

The first step of western blot quantification is measuring the band intensity of proteins of interest and an endogenous control (Fig. 1E). Various quantification programs, including ImageJ/Fiji and Bio-rad Image Lab (RRID: SCR_014210), are available for the measurement of band intensities on images (Kim et al., 2023a, Schindelin et al., 2012, Schneider et al., 2012). Upon completing the measurement, the levels of proteins of interest are normalized using the levels of an endogenous control protein (Varli et al., 2024). Endogenous control proteins often produce strong signals that can quickly saturate. Therefore, accurate normalization requires using nonsaturated bands. Proper antibody titration and comparison of multiple control proteins help ensure reliable reference selection (Pillai-Kastoori et al., 2020b, Taylor et al., 2013). This normalized value is used for comparison between samples and groups. Although quantification of western blot data is valuable for data analysis, researchers must interpret the results with caution because ECL signals are inherently nonlinear (Butler et al., 2019).

## CONCLUDING REMARKS

Here, we describe an overview of western blot analysis, including antibody choice, PAGE, protein transfer, visualization, stripping, and quantification of proteins (Table 1). Although we do not discuss detailed methods, this article will be useful to researchers who are not expertized in western blot assays by providing key considerations and resources.

**Table 1:** Summary of key considerations for western blot

Consideration	Options	Advantages
Primary antibody	Monoclonal	High specificity
	Polyclonal	High affinity

Secondary antibody	Chemiluminescence	High sensitivity
	Fluorescence	Multiplexed detection

Protein tagging	Fluorescent proteins	Detectable in live cells and animals
	Small peptide tags	Minimal size shifting

Polyacrylamide gel	Tris-glycine	Widely applicable
	Bis-Tris	Neutral pH, long shelf time of gels

Membrane material	Polyvinylidene fluoride	Durable and strong protein binding
	Nitrocellulose	Low background

Transfer method	Wet transfer	Optimal for large proteins
	Semidry transfer	Rapid and straightforward

Binning	Low	High resolution
	High	Strong signal

Stripping method	Mild	Minimal impacts on proteins
	Harsh	Complete removal of antibodies

## Funding and Support

This work was supported by KAIST Stem Cell Center (A0801080001) and the 10.13039/501100003725National Research Foundation of Korea (NRF) grant funded by the Korea government (10.13039/501100014188MSIT) (RS-2024-00408712) to S-J.V.L. and by NRF grants funded by MSIT (RS-2024-00337733 and RS-2025-14383304) to M-Y.K., who was also supported by the KAIST Grand Challenge 30 Project [KC30, N11240024].

## Author Contributions

**Seung-Jae V. Lee:** Conceptualization, Funding acquisition, Project administration, Supervision, Writing – review & editing. **Ji-Hoon Park:** Writing – review & editing. **Seokjun G. Ha:** Conceptualization, Visualization, Writing – original draft, Writing – review & editing. **Mi-Young Kim:** Writing – review & editing, Funding acquisition.

## Declaration of Competing Interests

The author Seung-Jae V. Lee is an Editor-in-Chief for *Molecules and Cells* and was not involved in the editorial review or the decision to publish this article.
